# Extended spectrum and metalo beta lactamase producing gram negative bacterial pathogens from cockroaches collected at hospital, Southern Ethiopia

**DOI:** 10.1186/s13756-024-01442-0

**Published:** 2024-08-13

**Authors:** Fithamlak Solomon Bisetegn, Habtamu Azene, Khawaja Shakeel Ahmed, Fiseha Wadilo, Efrata Girma Tufa

**Affiliations:** 1 School of Medical Laboratory, College of health sciences and Medicine, Wolaita Sodo University, Wolaita Sodo, P.O.Box: 138, Ethiopia; 2 School of Medicine, College of Health Sciences and Medicine, Wolaita Sodo University, Wolaita Sodo, Ethiopia; 3 Department of Medical laboratory sciences, Komar University of Science and Technology, Sulaymaniyah, Iraq; 4 Armauer Hansen Research Institute, Ministry of Health, Addis Ababa, Ethiopia; 5 Department of Biomedical Sciences, College of Natural and Computational Sciences, Addis Ababa University, Addis Ababa, Ethiopia; 6 School of Medical Laboratory Sciences, College of Health Sciences and Medicine, Wolaita Sodo University, Wolaita Sodo, Ethiopia; 7 School of public health, College of Health Sciences and Medicine, Wolaita Sodo University, Wolaita Sodo, Ethiopia

**Keywords:** Cockroach, Antibiotic, Resistance, ESBL, MBL, Hospital, Bacteria

## Abstract

**Background:**

Cockroaches can pose a significant health risk in hospital environments because they may serve as reservoirs and vectors for nosocomial pathogens. Cockroaches harbor epidemiologically significant extended spectrum and metalo beta lactamase producing Gram negative bacterial pathogens, which complicate nosocomial infections.

**Objectives:**

The main aim of this study is to determine aetiology and phenotypic extended spectrum and metalo beta lactamase producing Gram negative bacteria pathogens from cockroaches collected in hospitals.

**Methods:**

A cross-sectional study was employed from February to May 2022 to determine the antibiotic resistance producing bacterial isolates from cockroaches by giving special emphasis to metalo beta lactamase and extended spectrum beta lactamase production from different wards of WSUCSH. Cockroaches were collected with hands wearing sterile gloves. External homogenate was prepared and incubated microbiologically by using different culture media and differentiated biochemically. Antimicrobial susceptibility testing was performed by disk diffusion method. ESBL production was conducted using double disc synergy method and double disk method was used to detect MBL enzyme detection. Descriptive statistics was used to determine prevalence and percentage.

**Result:**

Out of 245 cockroaches, 108 Gram negative bacteria were isolated. *K. pneumoniae* 29(26.9%) was the most predominant bacteria and *Enetrobacter spp.* 8(7.4%), was the least. All, *K. pneumoniae*, *P. mirabilis*, and *Enterobacter* isolates were pan-resistant to Ampicillin. *P.aeruginosa* and *P.mirabilis* antibiotics showed ≥ 80% resistant for amoxicillin/clavulanic acid antibiotics. Cefotaxime, ceftazidime, ceftriaxone and imipenem showed relative efficacy compared with other antibiotics. Out of 78 amoxicillin-clavulanic acid resistant isolates, 42(34.7%) were ESBL producers. ESBL production is more depicted by *P. aeruginosa*,* A. baumannii*, *K. pneumoniae* and *E. coli*. The overall prevalence of MBL production is 29(23.1%). *K. pneumoniae P. aeruginosa*, *E.coli*, *A. baumannii*,* Enterobacter spp* and *K.oxytoca* revealed MBL production.

**Conclusion:**

The overall prevalence of ESBL and MBL producing nosocomial agents from hospital cockroaches was 34.7% and 23.1% respectively. *P.aeruginosa*,* A.baumannii*, *K.pneumoniae* and *E.coli* showed pronounced ESBL production. All bacterial isolates except *P. mirabilis* and *C. freundii* showed MBL production. The needed to evaluate our antibiotic stewardship program and antibiotic resistance detection for treatment is mandatory. The impact of cockroach as a source of AMR should be sought.

## Introduction

Antimicrobial resistance (AMR) is a serious global health threat that makes infections harder to treat and increases the risk of disease spread, severe illness and death. AMR is driven by the misuse and overuse of antimicrobials in humans, animals and plants. AMR was directly responsible for 1.27 million global deaths in 2019 and contributed to 4.95 million deaths. AMR also has significant economic costs and could result in US$ 1 trillion additional healthcare costs by 2050 [1].

Cockroaches are known to carry and transmit various bacterial species, including those that cause gastroenteritis in humans. They can contaminate food and food-handling surfaces through their droppings or by mechanical transfer from their bodies [2].

Cockroaches can pose a significant health risk in hospital environments because they may serve as reservoirs and vectors for nosocomial pathogens [3]. Cockroaches have long been regarded as possible vectors of human entero-pathogens owing to their unsanitary lifestyle and their indiscriminate feeding on sanitary wastes and human meals.

In hospitals, cockroaches can act as potential vectors in the epidemiology of nosocomial infections, especially the transmission of drug-resistant *Escherichia coli*, *Pseudomonas aeruginosa*, *Klebsiella* spp., and several other potential pathogens [3]. Studies have shown that 25 different species of medically important bacteria have been isolated from *cockroaches* in public hospitals in Ethiopia [5].

Cockroaches also harbor epidemiologically significant antibiotic-resistant organisms, such as carbapenem-resistant *Enterobacteriaceae*, which complicate nosocomial infections [3]. Studies in Ghana and Algeria showed that household and hospital cockroaches could serve as reservoirs of the *CTX-M-15*, *OXA-48*, and *NDM-1* genes that share beta-lactam resistance determinants with humans [6, 7]. The cockroach brain has antimicrobial properties, and this is thought to be an important factor that accounts for the carriage of antibiotic-resistant organisms among cockroaches [8].

In hospitals, cockroaches that carry antibiotic-resistant bacteria could easily disseminate these organisms on hospital equipment and, therefore, facilitate their transmission to patients. This implies that cockroaches could play a significant role in outbreaks of nosocomial pathogens in hospitals, though little attention has been given to this.

As a preliminary assessment, the hospital infection prevention team takes a visit to the hospital with our research team in 2021. Cockroach infestation was observed everywhere including patients’ meals provided by the hospital. Even though these pests are commonly available, no specific study was conducted on the insects as well no correlational intervention was also done on patients by assumption of how filthy these pests are. So this research team decided to conduct this study and reveal the findings to the hospital.

## Materials and methods

### Settings and population

A cross-sectional study was employed from February to May 2022 to determine the antibiotic resistance producing bacterial isolates by giving special emphasis to MBL and ESBL producing bacteria from wards of Intensive Care Unit (ICU), operation room (OR), Obstetrics (OBS), Out-patient department (OPD), Surgical (S) and Pediatric (P) wards where much cockroach infestation was observed in WSUCSH (Wolaita Sodo university comprehensive specialized teaching hospital). Wards were selected based on the patient flow, risk, longer hospital stay and availability of vulnerable patients who are at risk of hospital acquired infection.

Only adult cockroaches having whole body parts were included in the study and those cockroaches which were dead or showing missing body parts, nymphs, and eggs of cockroaches were excluded from further sample processing.

### Arthropod collection and sample preparation

A total of 245 cockroaches were randomly collected twice a day for 45 consecutive working days. The cockroaches were collected with hands wearing sterile gloves and placed in a sterile screw-capped 250 ml jar. Pests were transported to the WSUCSH microbiology laboratory for bacteriological analysis within five minutes of collection.

The collected cockroaches were immobilized by frigidity at 0°C for 5 min. The external body surface of immobilized cockroaches was washed by shaking in 5 ml of 0.85% sterile normal saline for two minutes and the wash was taken as an external homogenate sample and checked for bacterial growth [9, 10].

### Isolation and identification of bacterial pathogens

One ml of the external homogenates was suspended separately into 9 ml of sterile dilution test tubes containing buffered peptone water (BPW) and incubated at 37°C for 18–24 h. Each one of the growth from BPW was inoculated on the following primary media such as MacConkey agar, and sheep blood Agar for 18–48 h to grow. After 24 and 48 h pure colony of bacterial isolate was preliminary characterized by colony morphology, Gram-staining procedure, and API-20E Biomeriux France, for the isolation of *Eneterobacterciaeae* [10].

### Antimicrobial susceptibility testing

Antimicrobial susceptibility testing was performed for bacterial isolates by disk diffusion method on Mueller-Hinton agar (Oxoid). Bacterial inoculums were prepared by suspending the freshly grown bacteria in 4-5 ml sterile nutrient broth and the turbidity was adjusted to that of a 0.5 McFarland standard. The antimicrobial susceptibility testing was performed against the following discs, Amikacin (AMK, 30 µg); Ciprofloxacin (CIP, 5 µg); Gentamicin (GEN, 10 µg); Cefepime, CFP, 30 µg); Ampicillin (AMP, 10 µg); Amoxicillin/clavulanic acid (AMC 20 µg/10 µg; Ceftazidime (CAZ) 30 µg); Ceftriaxone (CRO, 30 µg) and Imipenem (IPM 10 µg). [11].

### Detection of ESBL production

ESBL production was conducted by using double disc method. Cefotaxime and ceftazidime resistant isolates under Kirby-Bauer disk diffusion test were selected and checked for ESBL. The bacterial suspension was prepared while taking 2–3 fresh colonies and adjusted to 0.5 McFarland standards. Lawn culture was done on the Mueller-Hinton Agar (MHA) plate. Cefotaxime (30 µg) and ceftazidime (30 µg) disks were placed onto the inoculated MHA plate and incubated overnight at 37°C. Isolates that revealed ≤ 22 mm inhibition zone size for ceftazidime and/or ≤ 27 mm for cefotaxime were considered as potential ESBL producers. The ceftazidime and ceftazidime/clavulanic acid discs and cefotaxime/ cefotaxime-clavuanic acid were placed at 20 mm apart on the agar surface and incubated overnight at 37°C. After overnight incubation a ≥ 5 mm increase in zone diameter for either cefotaxime or ceftazidime tested in combination with clavulanic acid, were taken as indicative for ESBL production [11].

### Detection of MBL production

Imipenem-resistant isolates were screened for producing MBL. The double disk method was used to detect this enzyme. A disc of Imipenem alone (10 µg) and Imipenem (10 µg) in combination with EDTA (750 µg/disc) was placed at the distance of 20 mm (center to center). After overnight incubation at 35 °C, a ≥ 7 mm increase in the inhibition zone of diameter around Imipenem-EDTA discs, as compared to imipenem discs alone, interpreted as indicative of MBL production [12].

### Quality control measures

The reliability of the study findings was guaranteed by implementing quality control measures throughout the whole process of laboratory work. Staining reagents, culture media, and antimicrobial discs were checked for their normal shelf life before use. Culture media were prepared based on the manufacturer’s instruction then its sterility was checked by incubating 5% of the batch at 35–37°C overnight and observing bacterial growth. The quality of culture media and antimicrobial susceptibility test was checked by using reference strains of *E. coli* (ATCC 25922) and *P. aeruginosa* (ATCC 6538). E. coli ATCC 25,922 and K. pneumoniae ATCC700603 (ESBL-positive isolate) were used as the negative and positive controls, respectively.

### Data processing and analysis

Data was edited and entered into SPSS for Windows version 20. Descriptive statistics was used to determine prevalence and percentage. The processed data were presented using a table and figure.

## Results

### Magnitude of cockroach infestation

**Fig. 1:**
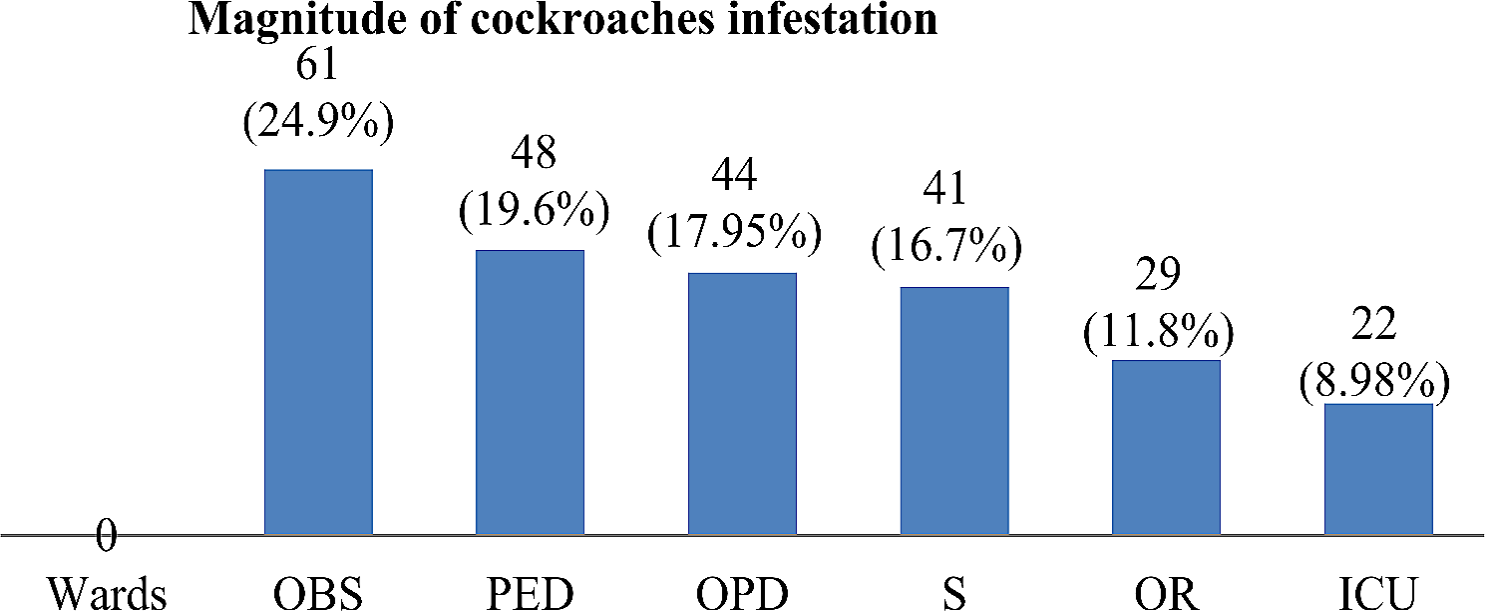
Magnitude of cockroach infestation in each ward of the hospital, Southern Ethiopia. 2022

Out of a total of 245 cockroaches, the highest infestations of cockroaches were seen in the obstetrics ward followed by the Pediatric ward, and OPD, Surgery, and OR ward followed by the prevalence of cockroaches respectively. However, the least infestation was recorded in the ICU room (Fig. 1).

### Bacteria isolated from Hospital cockroaches

**Fig. 2:**
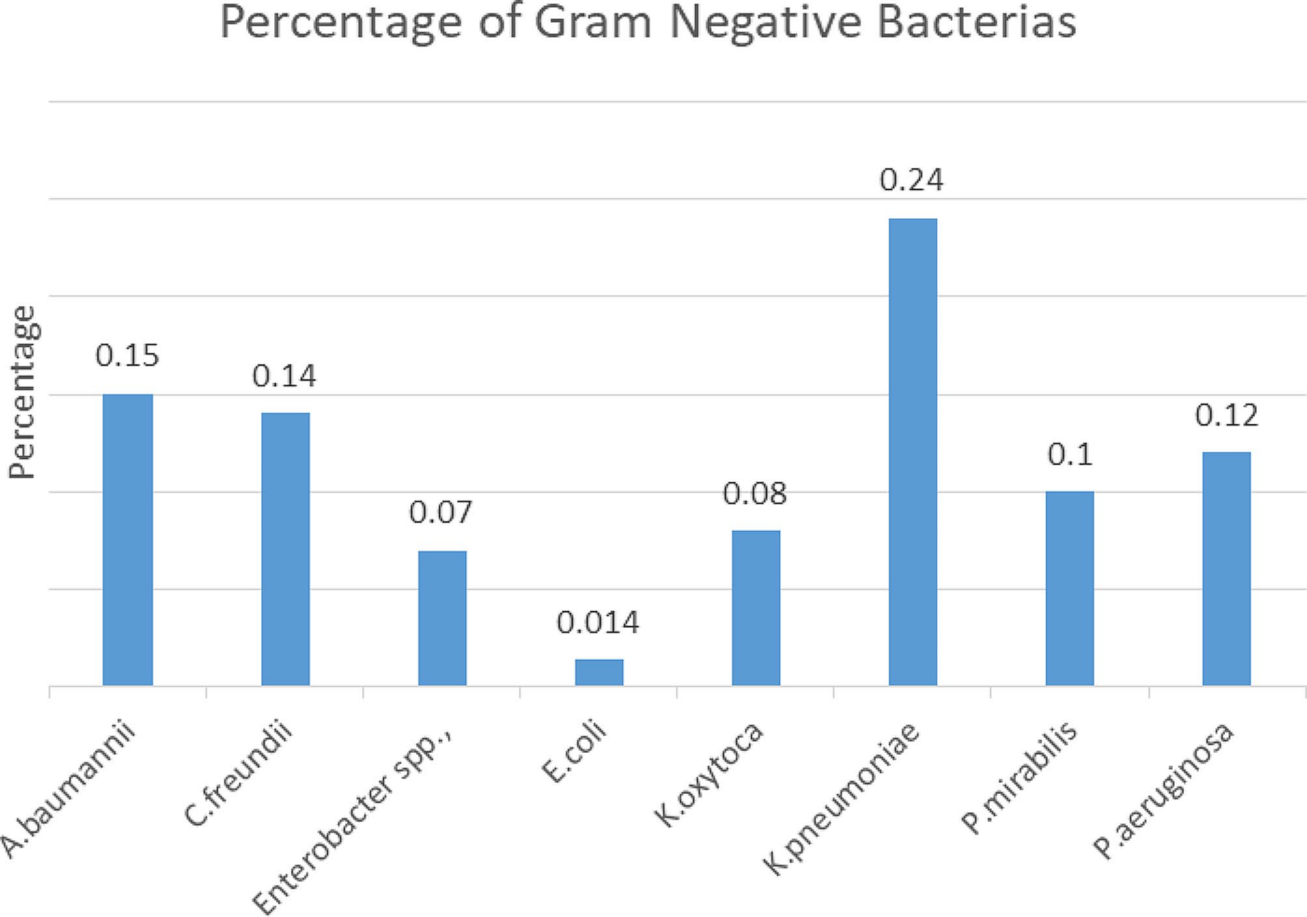
Medically important bacteria identified from cockroaches collected in the hospital, Southern Ethiopia, 2022

Out of 245 cockroaches collected in different wards of the hospital, 108 Gram negative bacterial isolates were isolated. *K. peumoniae* 29(26.9%) was the most predominant bacteria and *Enetrobacter* 8(7.4%), was the least (Fig. 2).

### Antimicrobial susceptibility pattern

The susceptibility pattern of bacterial isolates (*n* = 121) identified from cockroaches was tested for nine different antibiotics (Table 1). Accordingly, all *K. pneumoniae*, *P. mirabilis*, and *Enterobacter* isolates were pan-resistant to ampicillin. *P. aeruginosa* and *P. mirabilis* antibiotics showed ≥ 80% resistant for amoxicillin/clavulanic acid antibiotics. Cefotaxime, ceftazidime, ceftriaxone and imipenem showed relative efficacy compared with other antibiotics. *P. aeruginosa* depicted resistance rate of 66.7% for cefotaxime. *P.mirabilis* showed 66.7% resistance. Ceftazidime on the other hand showed > 60% resistance for *E. coli* and *Enterobacter spp. K. oxytoca* and *P. aeruginosa* both became resistant, 70% for imipenem. *A. baumannii* showed very high resistance for ciprofloxacin, and gentamicin (Table 1).

**Table 1:** Antimicrobial susceptibility pattern of Gram negative bacteria isolates and identified from cockroaches (*n* = 121) at WSUTRH, Southern Ethiopia. 2022

Antibiotics	K. pneumoniae *n* = 29	C. freundii *n* = 17	*P*. mirabilis *n* = 12	K.Oxytoca *n* = 10	*P*.aeruginosa *n* = 15	E. coli *n* = 17	Entrobacter *n* = 8	A.baumannii*n* = 13
AMP	S (%)	S (%)	S (%)	S (%)	S (%)	S (%)	S (%)	ND
	I (%)	I (%)	I (%)	I (%)	I (%)	I (%)	I (%)	
	R (%)	R (%)	R (%)	R (%)	R (%)	R (%)	R (%)	
	0(0)	4(23.5)	0(0)	0(0)	ND	0(0)	0(0)	0(0)
	0(0)	0(0)	0(0)	3(30)		2(28.6)	0(0)	0(0)
	29(100)	13(76.5)	12(100)	7(70)		15(71.4)	8(100)	13)
AMX/CLA	8(27.6)	5(29.4)	2(16.7)	0(0)	2(13.3)	5(29.4)	3(37.5)	4(30.8)
	0(0)	1(5.9)	0(0)	3(30)	1(6.7)	0(0)	0(0)	0(0)
	21(72.4)	11(64.7)	10(83.3)	7(70)	12(80)	12(70.6)	5(62.5)	9(69.2)
CFP	12(41.4)	10(58.8)	7(58.3)	5(50)	4(26.7)	7(41.2)	4(50)	5(38.5)
	3(10.3)	0(0)	0(0)	1(10)	1(6.7)	1(5.9)	0(0)	0(0)
	14(48.3)	7(41.2)	5(41.7)	4(40)	10(66.7)	9(52.9)	4(50)	8(61.5)
CAZ	15(58.8)	9(52.9)	8(66.7)	8(80)	6(40)	6(35.3)	3(37.5)	3(23.1)
	2(6.9)	0(0)	1(8.3)	0(0)	1(6.7)	0(0)	0(0)	1(7.7)
	12(41.4)	8(47.1)	3(25)	2(20)	8(53.3)	11(64.7)	5(62.5)	9(69.2)
CIP	2(6.9)	5(29.4)	4(33.3)	7(70)	0(0)	5(29.4)	0(0)	1(7.7)
	8(27.6)	3(17.6)	2(16.7)	0(0)	0(0)	2(11.8)	1(12.5)	0(0)
	19(65.5)	9(71.4)	6(50)	3(30)	15(100)	10(58.8)	7(87.5)	12(92.3)
CRO	15(51.7)	6(35.3)	4(33.3)	4(40)	ND	7(41.2)	5(62.5)	4(30.8)
	1(3.4)	3(17.6)	0(0)	0(0)		1(5.9)	0(0)	1(7.7)
	13(44.8)	8(47.1)	8(66.7)	6(60)		9(52.9)	3(37.5)	9(69.2)
AMK	0(0)	4(23.5)	3(25)	0(0)	2(13.3)	4(23.5)	0(0)	2)15.4)
	7(24.1)	1(5.9)	0(0)	2(20)	0(0)	0(0)	2(25)	1(7.7)
	22(75.9)	12(70.6)	9(75)	8(80)	13(86.7)	13(76.5)	6(75)	10(76.9)
GEN	0(0)	3(17.6)	2(16.7)	10(100)	2(13.3)	5(29.4)	0(0)	1(7.7)
	7(24.1)	1(5.9)	0(0)	0(0)	0(0)	1(5.9)	2(25)	1(7.7)
	22(75.9)	13(76.5)	10(83.3)	0(0)	13(86.7)	11(64.7)	6(75)	11(84.6)
IMP	10(34.5)	5(29.4)	7(58.3)	3(30)	4(26.7)	8(47.1)	5(62.5)	5(38.5)
	1(3.4)	1(5.9)	0(0)	0(0)	0(0)	1(5.9)	0(0)	0(0)
	18(62.1)	11(64.7)	5(41.7)	7(70)	11(73.3)	8(47.1)	3(37.5)	8(53.8)

### ESBL production

Out of 121 bacterial isolates, 42(34.7%) were ESBL producers. ESBL production is more depicted by *A.baumannii* 7(53.8%), *P.aeruginosa* 8(53.3%), and *K.pneumoniae* 15(51.7%)(Table 2).

**Table 2:** ESBL production among bacterial isolates collected from cockroaches in Hospital, 2022, South Ethiopia

Bacteria	NO (%)	Total
*A.baumannii*	7(53.8)	13
*C.freundii*	3(17.6)	17
*Enterobacter spp.*,	1(12.5)	8
*E.coli*	6(35.3)	17
*K.oxytoca*	3(30)	10
*K.pneumoniae*	15(51.7)	29
*P.mirabilis*	3(25)	12
*P.aeruginosa*	8(53.3)	15

### MBL production rate

**Fig. 3:**
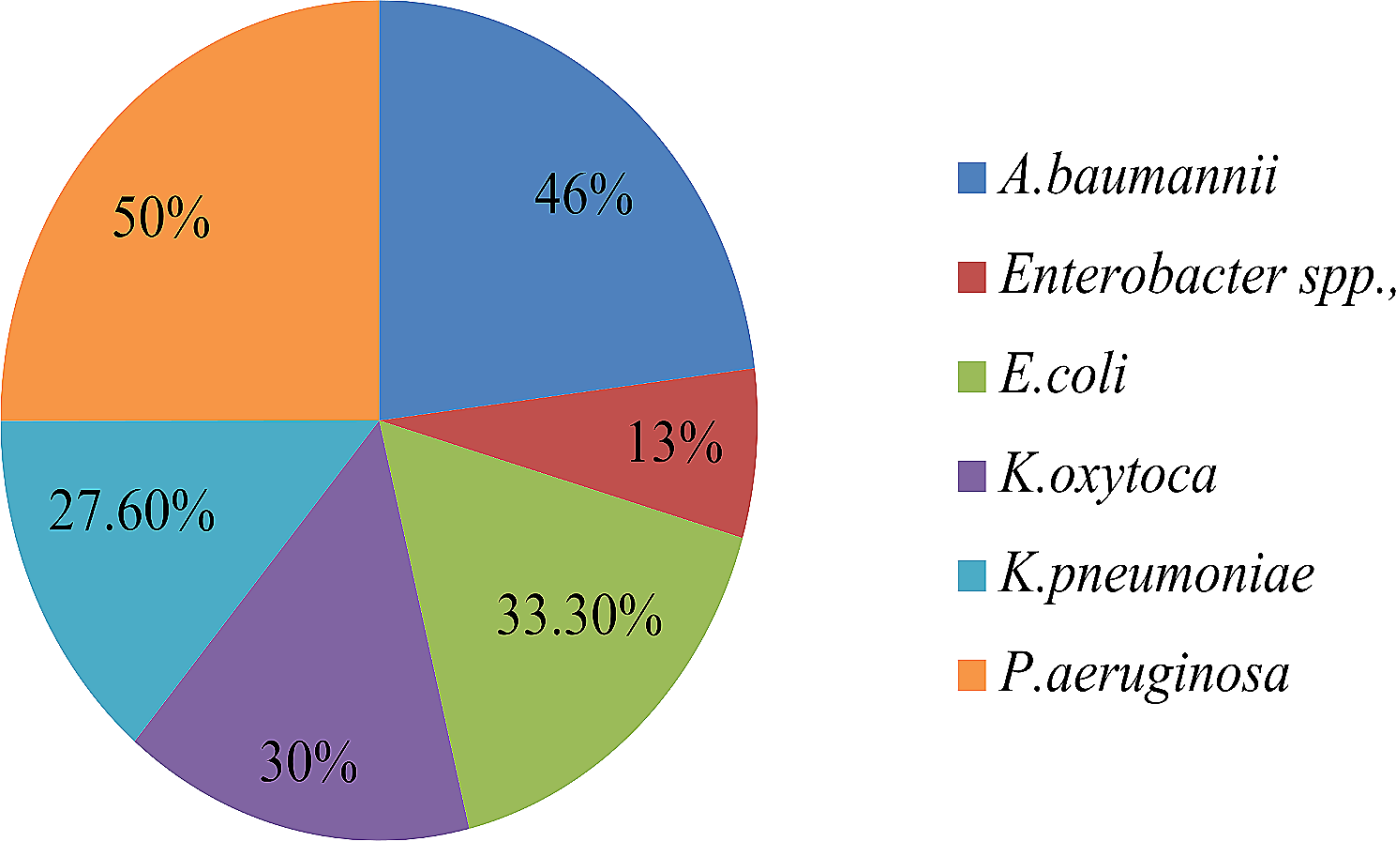
Metalo beta lactamase producing Gram negative bacteria pathogens isolates from cockroaches in the Hospital, 2022

The overall prevalence of MBL production is 28(23.1%). Half of the *P.aeruginosa* produces MBL and 6(46%) *A.baumannii* isolates were the second. On the other hand a single *Enterobacter* spp. revealed MBL enzyme production (Fig. 3).

## Discussion

Cockroaches as a reservoir for hospital acquired pathogens pose a serious concern in the hospital environment. Bacterial isolates of *A.baumannii*, *C. freundii*, *E. coli*, *Entrobacter*, *K.oxytoca*, *K. pneumoniae*, *P. mirabilis*, and *P.aeruginosa* were identified from cuticle of cockroaches in this study. Similarly, these bacteria were also isolated from external surface of cockroaches collected from health care settings according to different studies done previously [5, 13–21].

*K. pneumoniae* is the predominant isolate followed by *E.coli* and *C. freundii*. This finding is similar with a study conducted in the country and elsewhere [17, 22–25]. This isolates are responsible for diverse types of nosocomial and community acquired infections, notably pneumonia, urinary tract infection, respiratory tract infection, skin infections, septicemia, and gastroenteritis.

*A.baumannii* was first detected in the same setting from the hospital environment in the operation theatre and intensive care unit [26]. The detection of *A.baumannii* from cockroaches in different wards in this study is the first one compared with previous studies in the country. Previous studies detect *Acinetobacter* species but only to the genus level [5, 17]. On the other hand, previous studies elsewhere detected this bacterium from surface and gut of cockroaches in different environments. This may indicate that enough attention has not been given for this bacterium even though the World Health Organization (WHO) listed carbapenem-resistant *A. baumannii* as a critical priority, for which, new antibiotics are urgently needed [1].

Hospital cockroaches have been reported to carry highly antibiotic-resistant bacteria and they could easily disseminate on hospital equipment and facilitate their transmission to patients [3]. In the current study, very high rate of antibiotic resistance (≥ 80%) is evidenced by *P.aeruginosa (*AMX/CLA, CN, and AK), *A. baumannii* (AK, CN) *Enterobacter spp.* (AMP) and *P.mirabilis*(AMX/CLA). This finding corroborates with studies in the country and elsewhere [5, 7, 9, 16, 18, and 23]. Resistance rate which is in harmony with this study was also reported in the same setting from Hospital surfaces and inpatients where *P.aeruginosa*, *E.coli* and *Proteus* isolates become pan resistant for ampicillin [24, 25].

Most of the bacterial pathogens revealed high rate of cephalosporin (cefotaxime, ceftazidime and ceftriaxone) resistance in the current study. *E. coli* resistance to ceftazidime, and ceftriaxone was 80.5%, and 78.2%, respectively. Higher percentages of the isolates were also exhibited resistance to amoxicillin-clavulanic acid (74.1%), and cefoxitin (67.2%) in the previous study conducted from this pests [26] A cause for concern is the high resistance displayed to the cephalosporins, which are frontline antipseudomonal drugs for treating *P. aeruginosa* infections, increased resistance to this class of antibiotics will not be favorable and will result in limited treatment options [27].

*A.baumannii* showed high rate of resistance for ciprofloxacin, and gentamicin. Imipenem resistance for *A.baumannii* and *P.aeruginosa* was also observed from Hospital environment of operation and ICU of the same settings [28]. This antibiotics is one of the diagnostic indicator of MBL producers where > 70% resistance was also detected in different studies from cockroaches in the country and elsewhere. Resistance to carbapenem,70% to imipenem is quite unexpected, given the fact that carbapenems represent one of the most effective and among the best option for treating Gram-negative infections particularly MDR infections [29].

Vector potential of cockroaches for ESBL and MBL producing bacteria is a grave and worrisome concern. The overall prevalence of ESBL resistance is 34.7% in this study. Different prevalence of ESBL production was detected ranging from 15.6 to 91.7% across literature in the world. The discrepancy in different settings could be attributed to difference in bacterial isolates, study settings, antimicrobial stewardship program availability and number of isolate where percentages were formulated for MBL production [6, 7, 30–34].

In this study, all the isolated Gram negative bacterial isolates showed ESBL production. In the same manner, ESBL production among bacteria from cockroaches were also noted from previous findings elsewhere [6, 7, 30–37]. ≥50% ESBL production rate was revealed for *P.aeruginosa*, *A.baumannii*, *K. pneumoniae* and *E.coli* isolates. ESBL percentage of 55.8% and 62.5% was also isolated from *A. baumannii* and *P. aeruginosa* in the current settings from hospital environments [28].

The overall MBL production in this study is 23%. Studies corroborated with this finding, 19% were also evidenced from studies in Iraq [34]. Relatively comparable MBL production was detected from *P.aeruginosa and A.baumannii* isolates from environmental samples of the current study settings [28]. On the other hand, lower findings from ours, 13.8% [32] and 3% [6] were also recorded from Nigeria and Ghana respectively.

Five different bacterial isolates, *P.aeruginosa*, *A.baumannii*, *K.pneumoniae*, *E.coli* and *Enterobacter* species depicted MBL production in the current study. MBL producing bacterial isolates, *A.baumannii and P.aeruginosa* were also identified from cockroaches in Iran. In addition to that VIM-2 Metallo-β-Lactamase producing *Pseudomonas putida* was also detected from cockroaches in an Algerian Hospital for the first time. *E.coli* and *K.pneumoniae* MBL production from such pests were also detected from previous studies elsewhere [6, 7, 32–34,38]. *Enterobacter* species showing MBL production is the first of its own in the country though a study conducted in Algeria showed this finding [7].

## Conclusion

Extended spectrum beta lactamase and metalo beta lactamase production rate were 53.8% and 23.1% respectively. All identified bacterial isolates showed ESBL production. More than half of *P. aeruginosa A. baumannii K. pneumoniae*, isolates evidenced ESBL production. *A.baumannii*, *K.pneumoniae*, *E.coli* and *Enterobacter and K.oxytoca* isolates depicted MBL production with from 13 to 50%. Half of *P.aeruginosa* and 46% of *A.baumannii* depicted MBL enzyme production. Due attention is needed for non-clinical sources like cockroaches as a source for ESBL and MBL production. Antibiotic stewardship programs in the hospital needs to widen their target and take notice of vector capacity of cockroaches for AMR.

## Data Availability

No datasets were generated or analysed during the current study.

## Abbreviations

AMR: Antimicrobial resistance
ATCC: American type culture collection
BPW: Buffered Peptone Water
CLSI: Clinical and Laboratory Standards Institute
ESBL: Extended spectrum beta lactamase
ICU: Intensive Care Unit
MBL: Metalo beta lactamase
OPD: Out-patient department
OR: Operation room
OBS: Obstetrics
P: Pediatric
SPP: Species
SPSS: Statistical Package for Social Science
S: Surgical
WHO: World health organization
WSUCSH: Wolaita Sodo University comprehensive specialized Hospital
